# A diagnostic algorithm for inherited metabolic disorders using untargeted metabolomics

**DOI:** 10.1007/s11306-025-02302-7

**Published:** 2025-07-27

**Authors:** Qian Gao, Adnan Khan, Mette Christensen, Xiaomin Zhou, Allan Lund, Sabine Weller Grønborg, Flemming Wibrand, Elsebet Østergaard, Thomas Moritz

**Affiliations:** 1 Novo Nordisk Foundation Center for Basic Metabolic Research, University of Copenhagen, Copenhagen, Denmark; 2 Department of Clinical Genetics, Copenhagen University Hospital Rigshospitalet, Copenhagen, Denmark; 3 Center for Inherited Metabolic Diseases, Departments of Pediatrics and Adolescent Medicine and Clinical Genetics, Copenhagen University Hospital Rigshospitalet, Copenhagen, Denmark; 4 Department of Clinical Medicine, University of Copenhagen, Copenhagen, Denmark

**Keywords:** Metabolomics, Inherited metabolic disorders, Diagnosis, Unsupervised, IMD signature

## Abstract

**Introduction:**

Untargeted metabolomics is a powerful tool for detecting perturbations in biological systems, offering significant potential for screening for rare inherited metabolic disorders (IMDs). However, the rarity and vast diversity of these diseases, results in limited availability of samples and incomplete metabolic pathway knowledge for each condition. Current diagnostic procedures rely heavily on manual interpretation, which is time-consuming, and data driven approaches are insufficient for small sample sizes.

**Objectives:**

To develop a diagnostic algorithm for IMDs addressing the challenges posed by small sample sizes and continuously evolving datasets.

**Methods:**

77 IMD patients (35 different IMDs) and 136 control samples were collected from Copenhagen University Hospital, Rigshospitalet. The metabolome was analyzed using liquid chromatography-mass spectrometry. An algorithm partially based on sparse hierarchical clustering was designed to generate IMD-specific metabolic signatures from metabolomics data, enabling comparison with undiagnosed patient samples to provide diagnostic predictions. An iterative improvement strategy was employed, where new data are continuously integrated to refine the IMD-specific signatures. The algorithm’s performance was evaluated through both the current study and a case study using literature-derived data.

**Results:**

The algorithm demonstrated iterative improvement with each training round, correctly identifying the diagnosis within top 3 potential IMDs in 60% of samples (top 1 in 42%). The case study applied the method to literature-based data comprising 95 IMD samples (11 different IMDs) and 68 controls, yielding a correct diagnosis in 73.5% of cases.

**Conclusion:**

These results demonstrate that the algorithm provides a flexible, data-driven framework for continuous improvement in IMD diagnosis, even with limited number of samples.

**Supplementary Information:**

The online version contains supplementary material available at 10.1007/s11306-025-02302-7.

## Introduction

In recent decades, the number of identified inherited metabolic disorders (IMD) has grown rapidly. Timely and accurate diagnosis is crucial, as many IMDs are treatable, and early intervention can prevent irreversible damage.

Traditional biochemical diagnosis of IMDs can be complex and time-consuming, often involving repeated sampling, multiple sample preparation methods, and a range of analytical techniques. Even if whole genome sequencing (WGS) is part of a standard workup for IMD, it may not reliably identify the disease-causing variant(s), e.g. in case of a deep intronic variant or a variant of uncertain significance (VUS). This complexity can lead to diagnostic imprecision and may result in clinical deterioration before a final diagnosis is reached. Consequently, there is an urgent need for a more efficient IMD screening approach.

Metabolomics, the study of metabolites in a biological system, provides biochemical information that can be linked to specific phenotypes. Over the years, it has been adopted to identify and validate biomarkers for various IMDs. For example, targeted metabolomics has revealed 3-hydroxycaproic acid as a new biomarker in urine samples from patients with isovaleryl-CoA dehydrogenase deficiency, while also confirming established biomarkers like isovalerylcarnitine (Dercksen et al., 2013). Similarly, other studies have identified biomarkers for a broad spectrum of IMDs (Abela et al., 2017; Dénes et al., 2012; Smuts et al., 2013; Tebani et al., 2017; Venter et al., 2015). Additionally, metabolomics can be applied to various sample types, including plasma, urine, dried blood spots (DBS), and cerebrospinal fluid (CSF) (Haijes et al., 2019). This versatility enables its integration into scenarios ranging from newborn screening to routine blood checks, with minimal sample requirements.

Several computational methods have been developed for IMD screening based on untargeted metabolomics (Coene et al., 2018; Haijes et al., 2020; Thistlethwaite et al., 2022). However, a disadvantage with these approaches is that they typically require prior knowledge of metabolic pathways and good analytical coverage, or a large number of patient samples for each IMD. As a result, their performance is heavily dependent on the quality and quantity of the initial data. Optimal datasets are not easy to obtain in practice and can be particularly challenging for rare diseases like IMD, where the number of diseases is vast and patient data is sparse. In clinical settings, the slow and continuous collection of samples, along with variations in sample type, laboratory methods, and instrument performance, further complicates the diagnostic process.

To address these challenges, we present a diagnostic algorithm designed to work with small initial datasets while continuously incorporating new samples for iterative improvement. The algorithm is data-driven but allows for the integration of expert knowledge, making it flexible in adjusting the set of metabolites used for diagnosis as new data becomes available. By accommodating differences in metabolite coverage across various laboratories, our approach ensures robust and scalable IMD diagnosis. We demonstrate the value of this algorithm through tests on both experimental and literature-based data, highlighting its potential for routine clinical application.

## Method

### Sample collection

IMD EDTA-plasma samples were collected from a clinical biobank at Copenhagen University Hospital, Rigshospitalet. The biobank comprises leftover material from analyses performed in patients with a known or suspected IMD, and we included samples from all patients with a confirmed IMD diagnosis; this amounted to 77 IMD patients, representing 35 different IMDs, along with 136 controls (Table S1). IMD samples derived from patients already diagnosed with a specific IMD were sampled either (1) prior to initiation of treatment or (2) during treatment, when the biochemical profile specific to the IMD was still evident. Control samples originated from patients referred for diagnostic workup, but with no (proven) IMD. All samples from untreated patients had been collected in the period from 2005 to 2020; all samples from treated patients with IMD specific biochemical profile were collected autumn 2021 and all control samples were collected ultimo 2020. In all cases the samples had been stored at -20 °C during metabolic workup at the hospital, and at– 80 ℃ for long-term storage. Patients with an IMD were diagnosed based on detection of abnormal metabolites by sample analysis in the biochemical genetic laboratory and confirmed by molecular genetic testing (identification of likely pathogenic/pathogenic variant(s)). Median age in the untreated patient cohort was 5.5 years; 11.5 years in the treated patient cohort and 7.9 years in the control cohort (Table S4).

### Chemicals and reagents

High-performance liquid chromatography (HPLC)-grade water, acetonitrile, and methanol (MeOH) were purchased from Honeywell (Charlotte, NC, USA). Stable isotope labeled standards were acquired from Cambridge Isotope Laboratories, Inc. (Tewksbury, MA, USA). ESI-L Low Concentration Tune Mix was purchased from Agilent Technologies (Santa Clara, CA, USA). Formic acid 99.5+%, Optima™ LC/MS grade, was purchased from Fisher Chemical (PA, USA).

### Sample Preparation for metabolite extraction

For metabolomics analysis, sample preparation has been described elsewhere(Kronborg et al., 2024). Briefly, 100 µL of each plasma sample was aliquoted in 1.5 mL Eppendorf tubes and stored in a − 80 ℃ freezer until use. Metabolite extraction and protein precipitation was achieved by adding 395 µL of MeOH to the samples. For quality control and normalization, 5 µL of stable isotope labeled internal standards mix (10 µg/mL for all) containing L-carnitine-d3, octanoyl-L-carnitine-d3, butyryl L-carnitine-d3, palmitoyl L-carnitine-d3, uric acid-1,3-^15^N_2_, vitamin B5-^13^C_3_^15^N (Cambridge Isotope Laboratories, MA, USA), glutamine-^13^C_5_ (Sigma-Aldrich, MS, USA), glycoursodeoxycholic acid-d4, glycochenodeoxycholic acid-d4, taurolithocholic acid-d4, L- decanoic acid-d19, stearic acid-d35, decanoic acid-d19, and oleic Acid-d17 (Cayman chemicals, MI, USA) in MeOH was added to the extraction solvent. Samples were vortexed and kept on ice for 30 min. Thereafter the extracts were centrifuged at 18,000×*g* at 4 ℃ for 15 min for protein precipitation and metabolite extraction. The supernatant (320 µL) containing the polar metabolites was collected in new 1.5 mL Eppendorf tubes and dried under N_2_ (stream flow = 6 L/min) for 1 h at room temperature. The dried extract was reconstituted with 50 µL MeOH and 50 µL LC-MS-grade water further and analysed as described below.

### Quality control

Pooled samples dedicated for quality control (QC), were collected by mixing equal volumes of each sample. The pooled sample was further diluted with MeOH at 1:1, 1:2 and 1:3. To condition the column, the QC sample dilution series were injected at least 3 times before initiating the analytical run. The QC sample dilution series was reinjected every 20 sample injections, and at the end of the run to assess instrument stability and analyte reproducibility. An equal volume of a blank sample consisting of 100% MeOH was randomly inserted among the real sample queue to be processed as a needle wash and to equilibrate the column, as well as to avoid contamination among real samples. The analytical reproducibility in terms of detected intensities of detected *m/z* features was evaluated by calculating the coefficient of variation (% CV) of detected peaks in QC samples and by visualizing the tight clustering of QC samples in principle component analysis (PCA)(Vorkas et al., 2015). Specifically, only peaks satisfying the following criteria are retained: (1) retention time CV below 2%; (2) mass deviation within ± 5 ppm; and (3) peak area CV below 20%.

### Analysis of metabolites by UHPLC-TOFMS

Data acquisition was performed with an ultra-high-performance liquid chromatography quadrupole time-of-flight mass spectrometer (UHPLC-QTOFMS). A 1290 Infinity II LC System (Agilent Technologies, Waldbronn, Germany) was coupled to a timsTOF Pro mass spectrometer (Bruker Daltonics, Bremen, Germany) for reverse phase (RP) based metabolomics or a Bruker Impacts II QTOF mass spectrometer for Hydrophilic Interaction Chromatography (Hilic) based metabolomics. RP separations were performed on an Acquity UPLC HSS T3 Column, 100Å, 1.8 μm, 2.1 mm X 50 mm (Waters, Milford, MA). The column and auto-sampler temperatures were maintained at 40 °C and 10 °C, respectively. Solvent A consisting of 0.1% formic acid in water and solvent B consisting of 0.1% formic acid in acetonitrile and propanol (3:1, v/v), were used as mobile phases. The injection volume and flow rate were 2 µL and 0.4 mL/min, respectively. The UPLC gradient was programmed as follows: 3-100% B over 0–9 min, 100% B (9–14 min), 100-3% B (14–14.5 min) and 3% B (14.5–17 min). HILIC separations was achieved using an Atlantis Premier BEH Z-HILIC Column, 1.7 μm, 2.1 mm X 100 mm (Waters, Milford, MA). The column and auto-sampler temperatures were maintained at 50 °C and 10 °C, respectively. Solvent A consisting of 10 mM ammonium acetate in water spiked with 1 mL of 25% ammonium hydroxide per L and solvent B consisting of 10 mM ammonium acetate in 90% acetonitrile spiked with 3 mL of 25% ammonium hydroxide per L, were used as mobile phases. The injection volume and flow rate were 2 µL and 0.35 mL/min, respectively. The UPLC gradient was programmed as follows: 95 − 30% B over 0–7 min, 30–95% B (9–9.5 min) and 95% B (9.5–14 min). RP-MS analysis were performed in both positive ion mode and negative ion mode using a Vacuum Insulated Probe Heated ElectroSpray Ionization (VIP-HESI) ion source. The VIP-HESI parameters were as follows: 4500 V capillary voltage, 10.0 L/min dry gas, and temperature 230 ℃, probe gas flow 5.0 L/min and temperature 450 ℃. For Hilic-MS analysis an electrospray ionization (ESI) source was used. The ESI source parameters were as follows: 3600 V capillary voltage, 10.0 L/min dry gas, and temperature 230 ℃. The scan range was 50–1000 m/z at a scan speed of 2 Hz for both RP- and Hilic-MS analyses, and the resolution was approximately 60,000. For mass calibration, 50 µL of internal calibrant of 1 mM Na-formate was injected at the beginning of each analysis.

### MS-data processing for metabolomics analysis

Data acquisition was performed with Otof Control (version 6.3) and Compass HyStar (version 6.0. 30. 0) (Bruker Daltonics, Bremen, Germany), and data preprocessing was performed with Metaboscape 2021b software (Bruker Daltonics, Bremen, Germany). A total of 11,388 molecular features from reverse phase positive mode, 6,535 from reverse phase negative mode, and 1,722 from HILIC negative mode were detected and annotated in a two-step process. First masses and retention times were compared with an in-house library based on authentic standards. Secondly, the recorded MS/MS spectra in MetaboScape were annotated using SmartFormula and by comparing with the in-house MS/MS spectral library, Bruker HMDB Metabolite Library, Bruker MetaboBASE Personal Library 3.0, MoNA, and MSDIAL-TandemMassSpectralAtlas. A parent mass tolerance of 5 mDa was applied, with a SmartFormula mass error threshold of 5 ppm and a minimum MS/MS score of 600. In total, 135 features were annotated with Level 1 identification according to the Metabolomics Standards Initiative (MSI) guidelines, indicating confirmation by authentic standards based on matching retention time, exact mass, and MS/MS spectra (Table S5). Then features from different analytical methods was normalized separately against the internal standard which resulted in the lowest coefficient of variation of them in pooled QC samples. Missing values were imputed as the limit of detection.

### Diagnostic algorithm

The overall algorithm aims to define a set of metabolites and corresponding scores for each IMD, referred to as the “IMD signature”, which serve as potential diagnostic indicators rather than confirmed biomarkers. These signatures act as a reference for comparing with undiagnosed patient samples, where the similarity between the undiagnosed sample and IMD signatures indicates its resemblance to various IMD types.

**Fig. 1:**
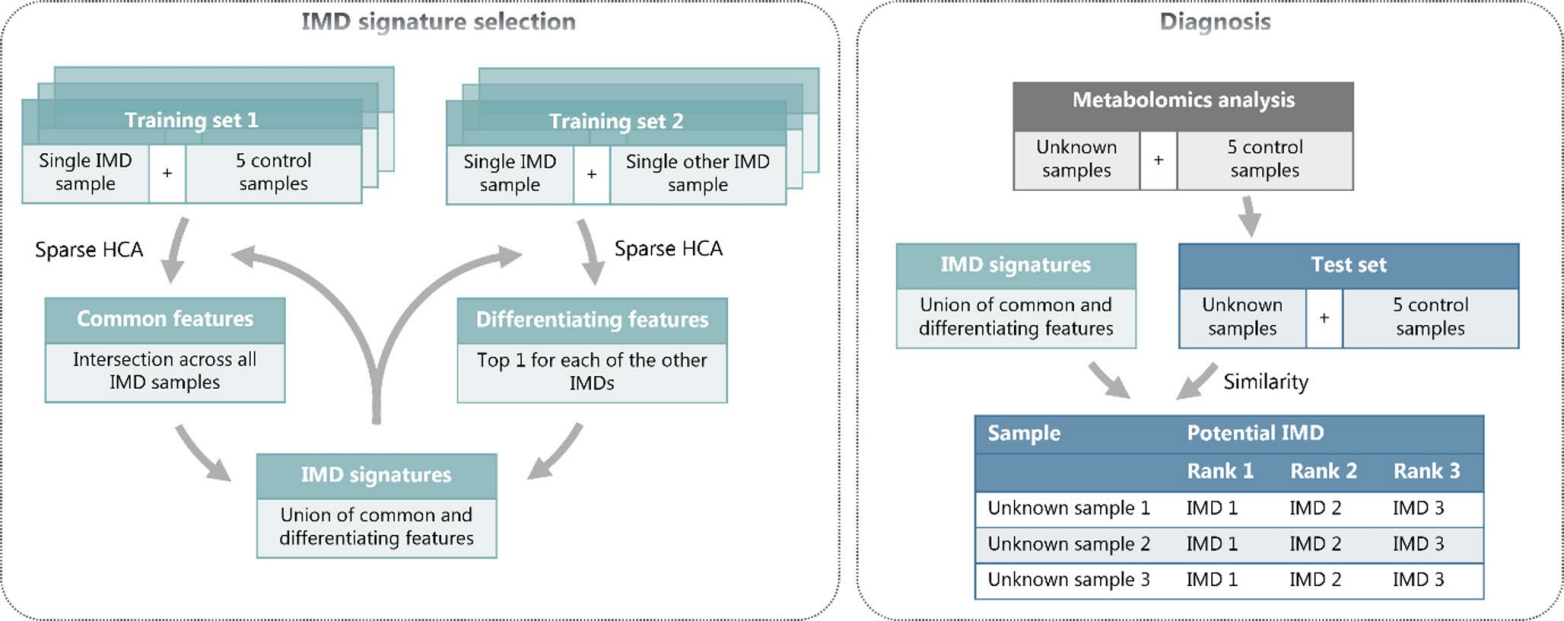
Workflow of the diagnostic algorithm

The diagnostic procedure is divided into three phases (Fig. 1):

#### Phase 1: discovery of IMD signatures

For each target IMD, two groups of training sets are created. The Training set 1 consists of a target IMD patient sample paired with five control samples, designed to identify metabolites that differentiate between the target IMD patient and the control population. The Training set 2 includes one target IMD patient sample and one sample from a different IMD type, aimed at finding metabolites that distinguish the target IMD from other IMDs.

For each training set, metabolite intensities are standardized as Z-scores, calculated based on the mean and standard deviation of the control samples. Sparse hierarchical clustering (Sparse HCA) (Witten & Tibshirani, 2010) is then applied to assign weights to each metabolite, reflecting its contribution to discriminating between samples. In the first group of training sets, the top 20 metabolites with the highest weights are selected from each set, and their intersection across all sets forms the metabolic signature for the target IMD. In the second group of training sets, the single top-weight metabolite from each set is selected, and their union is taken as part of the target IMD signature. The final IMD signature is the union of metabolites selected from both group of training sets, with the mean Z-score used as the final reference score.

#### Phase 2: refinement of IMD signatures

Refinement of IMD signatures is performed by metabolomic analysis of IMD samples not included in phase 1. The process from phase 1 is then repeated to generate an updated signature for the target IMD. In Training set 2, other IMD samples can be either new IMD samples or the existing IMD signatures. The union of metabolites from the old and new signatures is retained, and their respective scores are averaged to produce an updated reference score for the target IMD. This process can be repeated multiple times when new patient samples are available to improve the precision and robustness of the algorithm.

#### Phase 3: diagnosis using IMD signatures

For a patient sample with undiagnosed IMD, metabolomic analysis is performed together with five control samples, and the resulting data are standardized as described in phase 1. The undiagnosed sample is then compared to all IMD signatures across different IMDs. A normalized dot product similarity score is calculated for each comparison, indicating the degree of resemblance between the metabolic profile of the undiagnosed sample and the various IMDs. The similarity scores from all comparisons are then sorted in descending order, and the top 3 IMDs (denoted as Rank 1–3 diagnosis) are considered the most probable diagnosis for the patient sample.

## Case studies

### IMD samples from Copenhagen university hospital, Rigshospitalet

The diagnostic algorithm was tested on the IMD samples from Copenhagen University Hospital, Rigshospitalet, which is described in the sample collection section. In the first phase, IMD signatures were generated from 35 samples from IMD patients, each corresponding to a unique IMD. During the second phase, further training was conducted on IMDs with sufficient diagnostic samples available, including samples obtained during treatment but still with an IMD-specific biochemical profile. Refinement of the signatures using patient samples were possible for four specific IMDs, which have multiple samples available: Maple Syrup Urine Disease (MSUD), Medium-Chain Acyl-CoA Dehydrogenase Deficiency (MCADD), Methylmalonic Aciduria (MMA), and Isovaleryl-CoA Dehydrogenase Deficiency (IVDD). Three rounds of improvement were applied for MSUD, MCADD and MMA, while two rounds were conducted for IVDD.

### IMD data from literature

Given the limited availability of IMD patient samples in this study, the algorithm was also applied to data from a published study (Thistlethwaite et al., 2022), for further evaluation. The published dataset consisted of 95 IMD samples (11 IMDs) and 68 controls. In the first phase, signatures were identified for 11 IMDs. During the second phase, three rounds of improvement were performed to refine the signatures.

The algorithm was applied to the two case studies independently without cross-refinement due to inconsistent metabolite naming between them. In the third phase of both case studies, the IMD samples that had not been used for training were employed to assess diagnostic performance (31 sample for Case study 1 and 57 samples for Case study 2). A cross-validation scheme with 100 repetitions of repeated random sub-sampling was utilized (Table S2). In each iteration, samples were randomly selected within each category (control and various IMD subtypes), allowing the same samples to be included in different phases across iterations. This approach ensured a robust and unbiased evaluation of the algorithm’s diagnostic performance.

### Evaluation

To assess the performance of the algorithm, the top three most probable diagnosis (Rank 1–3 diagnosis) were compared against the true diagnosis for each IMD subtype. The number of correct diagnoses falling into each rank was recorded. Given that the entire procedure was repeated 100 times using cross-validation, standard deviations were calculated and reported to evaluate the robustness and consistency of the diagnostic performance.

## Results

### Case study 1

A total of 213 samples were analyzed with LC-MS, including 136 control samples and 77 patient samples across 35 IMDs. Twelve of the IMDs had multiple patient samples. The IMD signatures were discovered and refined on 66 samples (46 IMD and 20 control samples), and the diagnostic tests were conducted on 36 samples (31 samples representing the 12 IMDs with multiple samples and 5 control samples), as specified in the cross-validation scheme (Table S2). For each test sample, the top 3 most probable diagnosis (Rank 1–3) were compared against the true diagnosis of the test sample. The diagnostic outcomes are summarized in Table 1.

**Table 1:** Diagnosis for 31 samples in case study 1

	Rank 1	Rank 2	Rank 3	Total (Rank 1–3)
Initial	11.2 (3.7)	3.1 (1.6)	2.1 (1.4)	16.4 (4.3)
1st iteration	12.4 (3.9)	3.3 (1.7)	1.9 (1.3)	17.6 (4.3)
2nd iteration	12.9 (4)	3.3 (1.7)	1.8 (1.2)	18 (4.2)
3rd iteration	13.2 (4)	3.4 (1.7)	2 (1.4)	18.6 (4.1)

Data is presented as the number of samples correctly diagnosed per iteration and diagnostic rank, with standard deviation in parentheses. The diagnostic rank reflects the similarity between the undiagnosed sample and IMD signatures, with a higher rank indicating higher similarity and a higher probability of an accurate diagnosis

The diagnostic test was performed on the initial IMD signatures and also the refined IMD signatures after every iteration to track the progress of the effectiveness of the IMD signatures on diagnosis. After the initial training phase, approximately half of the samples (16.4 of 31) were correctly diagnosed within the top 3 rankings. With each iteration, the number of correct diagnoses increased. However, the efficacy of the algorithm differs in different IMD. Table 2 illustrates the diagnostic rates for individual IMDs, where most had a diagnostic rate exceeding 50%. While certain IMD, such as Holocarboxylase synthase deficiency (HLCSD) and 2-hydroxyglutaric aciduria, had notably lower diagnostic rates.

**Table 2:** Diagnosis for different IMDs in case study 1

IMD	Total number of samples	Number of test samples	Rank 1	Rank 2	Rank 3	Total (Rank 1–3)
Medium chain acyl-CoA dehydrogenase deficiency (MCADD; MIM#201450)	17	13	5 (3.2)	1.2 (1.2)	0.7 (0.9)	6.9 (3.9)
Maple syrup urine disease (MSUD; MIM#248600, 620699)	7	3	2 (0.9)	0.4 (0.6)	0.2 (0.5)	2.6 (0.7)
Methylmalonic aciduria (MMA; MIM#251000, 251110)	7	3	1.4 (0.9)	0.5 (0.6)	0.2 (0.5)	2.2 (0.8)
Isovaleryl-CoA dehydrogenase deficiency (IVDD: MIM#243500)	5	2	1.8 (0.5)	0.1 (0.3)	0 (0.2)	1.9 (0.3)
Holocarboxylase synthase deficiency (HLCSD; MIM#253270)	3	2	0 (0.1)	0 (0.1)	0 (0.2)	0.1 (0.3)
Peroxisome biogenesis disorders (several MIMs)	3	2	1.1 (0.9)	0.4 (0.6)	0.2 (0.4)	1.7 (0.6)
D-2-hydroxyglutaric aciduria (MIM#600721)	2	1	0 (0)	0 (0)	0 (0)	0 (0)
Argininosuccinic aciduria (MIM#207900)	2	1	0.3 (0.5)	0.3 (0.5)	0.1 (0.3)	0.8 (0.4)
Carnitine transporter deficiency (CTD; MIM#212140)	2	1	0.1 (0.3)	0.2 (0.4)	0.2 (0.4)	0.5 (0.5)
Lysinuric protein intolerance (MIM#222700)	2	1	0.8 (0.4)	0.1 (0.3)	0 (0.2)	1 (0)
Malonyl-CoA decarboxylase deficiency (MIM#248360)	2	1	0.5 (0.5)	0.2 (0.4)	0.1 (0.3)	0.8 (0.4)
Nonketotic hyperglycinemia (MIM#605899)	2	1	0.1 (0.3)	0 (0.2)	0.1 (0.3)	0.2 (0.4)

For each IMD, the table lists the number of samples collected in the study and used for diagnostic testing. The diagnosis results are presented as the number of samples correctly diagnosed per diagnostic rank after three iterations of refinement, with standard deviation in parentheses. The diagnostic rank reflects the similarity between the undiagnosed sample and IMD signatures (Table S6), with a higher rank indicating higher similarity and a higher probability of an accurate diagnosis

**Fig. 2:**
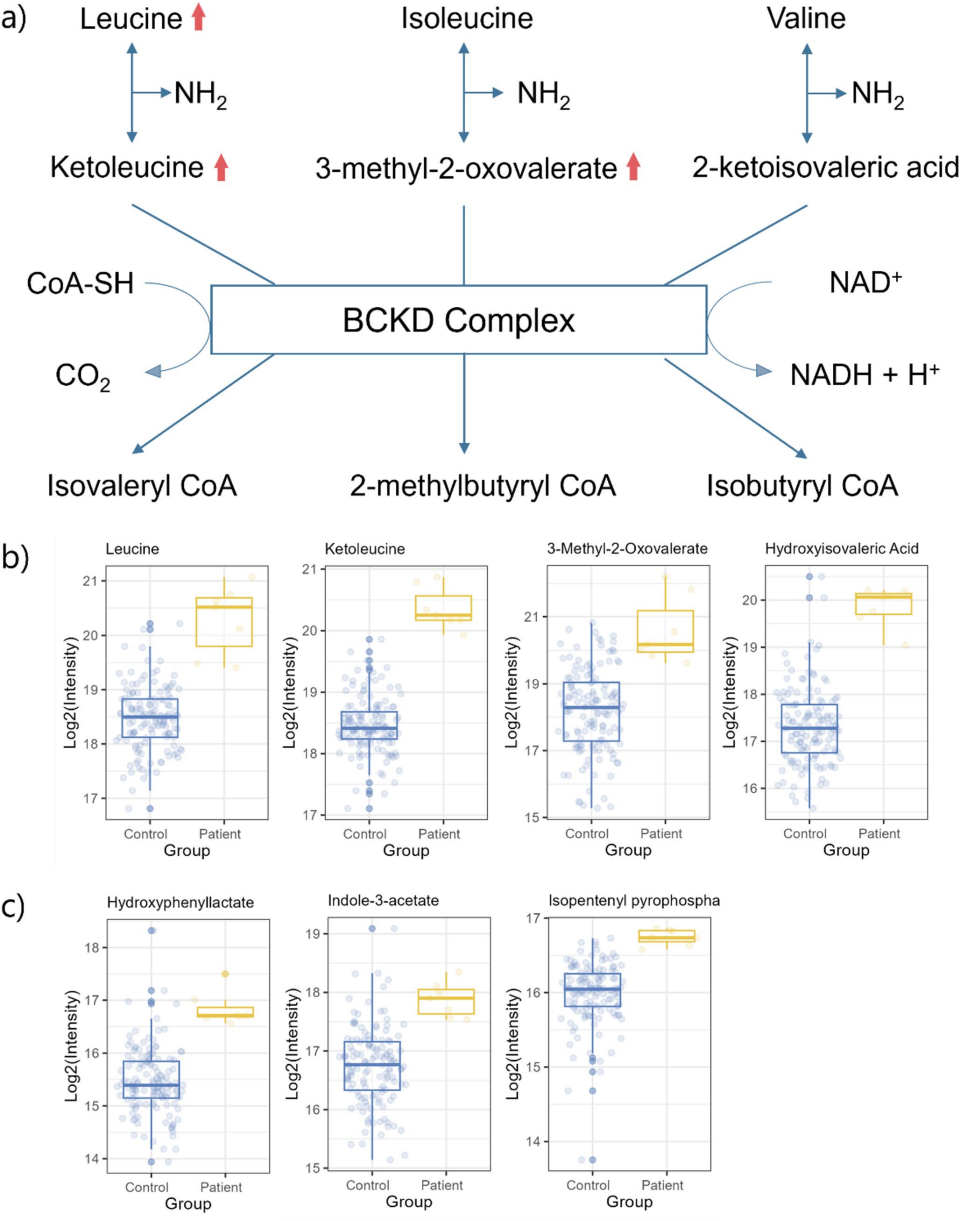
**a** Involved pathway; **b** Established biomarkers; and **c** Potential biomarkers for Maple Syrup Urine Disease (MSUD)

When analyzing the metabolites selected by the algorithm, well-established biomarkers were found in the IMD signatures alongside potential new ones. For example, in MSUD, the deficient or decreased activity of the BCKD complex leads to the accumulation of branched-chain amino acids, as reflected by elevated levels of leucine, ketoleucine, and their downstream metabolites (3-hydroxyisovaleric acid and 3-methyl-2-oxovalerate) (Fig. 2). Additionally, metabolites such as hydroxyphenyllactate (tyrosine metabolite), indole-3-acetate (tryptophan metabolite), and isopentenyl pyrophosphate were identified, showing increased levels in MSUD patient samples. These findings suggest they could serve as potential novel biomarkers for MSUD. However, it is important to note that all MSUD samples analyzed were obtained during treatment (branched-chain amino acid restriction). Consequently, the elevated levels of tyrosine and tryptophan-derived metabolites may be influenced by dietary intervention rather than the disease process alone.

### Case study 2

In the second case study, 58 samples (38 IMD and 20 control samples) were used for IMD signatures discovery and 3 iterations of refinement (Table S2). Then the diagnostic tests were applied to diagnose 57 undiagnosed patient samples with 5 control samples added. The diagnostic results are presented in Table 3, showing the algorithm’s performance across multiple iterations.

**Table 3:** Diagnosis for 57 samples in case study 2

	Rank 1	Rank 2	Rank 3	Total
Initial	41.9 (3.7)	5.8 (2.5)	1.9 (1.4)	49.6 (4)
1st iteration	41.5 (10.5)	4.3 (2.2)	1.5 (1.3)	47.3 (11)
2nd iteration	42.8 (10.8)	3.5 (1.8)	1.2 (1.3)	47.5 (11)
3rd iteration	43.5 (10.6)	3 (1.6)	1 (1.2)	47.6 (10.8)

Data is presented as the number of samples correctly diagnosed per iteration and diagnostic rank, with standard deviation in parentheses. The diagnostic rank reflects the similarity between the undiagnosed sample and IMD signatures, with a higher rank indicating higher similarity and a higher probability of an accurate diagnosis

The initial diagnostic accuracy in case study 2 was around 87%, with approximately 73.5% of samples correctly identified at Rank 1. After several iterations of training, the total number of correct top diagnoses decreased slightly; However, the proportion of correct Rank 1 diagnoses improved to 76.3% (43.5 of 57). Similar to Case study 1, MSUD and MMA exhibited strong diagnostic performance, with a correct diagnostic rate at or above Rank 3 exceeding 90% (with 90% for MSUD and 80% for MMA at Rank 1, Table 4).

**Table 4:** Diagnosis for different IMDs in case study 2

IMD	Total number of samples	Number of test samples	Rank 1	Rank 2	Rank 3	Total (Rank 1–3)
Maple syrup urine disease (MSUD; MIM#248600)	18	14	13.1 (3.3)	0 (0.2)	0 (0.1)	13.2 (3.3)
Ornithine transcarbamylase deficiency (MIM#311250)	17	13	7.1 (3.9)	1.4 (1.3)	0.7 (1)	9.1 (3.8)
Citrullinemia (MIM#215700)	9	5	4.7 (1.1)	0.1 (0.3)	0 (0)	4.8 (1.1)
Methylmalonic aciduria (MMA; MIM#251100, 251000)	9	5	4.1 (1.4)	0.6 (0.9)	0 (0)	4.7 (1.2)
Propionic acidemia (MIM#606054)	9	5	4.7 (1.3)	0 (0)	0 (0)	4.7 (1.3)
Cerebral creatine deficiency syndrome 2 (MIM#612736)	8	4	1.1 (0.9)	0.2 (0.4)	0.3 (0.5)	1.6 (1)
Phenylketonuria (MIM#261600)	8	4	3.7 (1.1)	0 (0.2)	0 (0.2)	3.8 (0.9)
Cobalamin biosynthesis defect (MIM#277400, 277410, 236270, 277380, 250940, 614857, 309541)	6	2	1.1 (0.8)	0.7 (0.7)	0 (0.2)	1.8 (0.6)
Glutaric acidemia 1 (MIM#231670)	5	2	2 (0)	0 (0)	0 (0)	2 (0)
Argininemia (MIM#207800)	4	2	2 (0)	0 (0)	0 (0)	2 (0)
Argininosuccinic aciduria (MIM#207900)	2	1	0 (0.1)	0 (0.2)	0 (0.2)	0.1 (0.3)

## Discussion

### Continuous improvement through iterative training

A key advantage of this algorithm is its ability to improve iteratively with new data. Traditional data-driven methods often rely on having a large set of training samples from the outset to establish metabolic signatures for diagnosis. While beneficial, such an approach is often impractical for rare diseases like IMD, where sample sizes are limited. Our algorithm is designed to handle small initial datasets and incorporates new samples iteratively for continuous refinement. To mitigate the influence of outliers, both analytical and biological, reference scores from earlier iterations are assigned higher weights, ensuring that the core metabolic features remain stable while the algorithm gradually incorporates new features. After large number of iterations, the variabilities would be averaged out, the possible analytical error would be gradually eliminated, the IMD signatures incline to represent the average IMD metabolic profile. This allows the system to refine its diagnostic accuracy over time, reaching a relatively stable performance after enough iterations.

Given the small sample size, we employed an unsupervised approach to reduce the risk of overfitting and reduced generalizability. One inherent limitation of unsupervised methods is that they do not learn directly from labeled data, which can make it difficult to distinguish true disease patterns from background noise. However, through iterative learning, the algorithm becomes progressively more robust. Important features are reinforced across repetitions, while less informative or noisy variables are gradually phased out. This enables the model to improve its diagnostic precision over time, even in the absence of large, labeled training sets.

### Scalability for new IMD types and different metabolites set

The number of IMDs included in the diagnostic process significantly impacts performance, particularly when IMDs have similar metabolic profiles. Therefore, expanding the algorithm’s capacity to accommodate newly discovered IMDs is critical for improving its ability to differentiate between conditions. Additionally, the algorithm’s performance depends on the metabolite coverage provided by the training set. While the algorithm can operate with incomplete metabolite measurements, biased coverage—where certain IMDs have more comprehensive metabolite data than others—may skew the diagnostic results. IMDs with more complete metabolite data are more likely to achieve higher diagnostic accuracy, while those with less data may be underdiagnosed. Our algorithm is designed to scale efficiently with new IMDs and metabolites. Newly detected metabolites are initially included with small weights, allowing the model to integrate them gradually into the diagnostic process as additional data becomes available.

### Objectivity and the role of expert judgment

The developed algorithm ranks and selects metabolites for diagnosis without expert input, making it suitable for both well-characterized and less-studied IMDs. It also has the potential to generate new hypotheses for underexplored metabolic profiles. Unlike pathway-based diagnostic methods that rely heavily on prior knowledge (Coene et al., 2018; Haijes et al., 2020), our approach is more flexible, providing consistent diagnostic predictions even for IMDs lacking clear pathway information. However, the algorithm allows for expert input, where users can adjust the weighting of specific metabolites based on clinical experience. For example, in Case Study 1, methylmalonic aciduria (MMA) and homocystinuria (CblC type), not surprisingly shared five metabolic signatures with MMAs without homocystinuria– 3-hydroxyphenylacetic acid, methylmalonylcarnitine, succinic acid, 2-methylbutyrylcarnitine and proline. If an undiagnosed sample receives the top two diagnoses for MMAs, a second-round diagnostic procedure can be conducted to identify if a subtype of MMA can be identified for this sample. In this subsequent round, the weights of the five shared signatures can be reduced, while increasing the weights of other distinguishing signatures. This approach would specifically target the differences between these two IMDs, leading to a more confident and accurate diagnosis. This flexibility provides a valuable tool for refining the model based on expert judgment, especially for IMDs with well-characterized pathways or biomarkers.

### Influence of treated samples

One factor contributing to lower diagnostic accuracy is the heterogeneity of the training samples. The algorithm relies on specific features selected from the training set to diagnose new samples. Samples obtained from treated individuals may deviate from the typical IMD pattern found in samples from untreated individuals. This may lead to selection of ambiguous features, and thus reduced diagnostic accuracy. Maintaining a high similarity between the training samples is therefore crucial when selecting them for training. In our case study, where testing involved both treated and untreated samples, including treated samples in the training process helped capture a more representative metabolic pattern, as reflected in the initial diagnosis. In contrast, training exclusively on untreated samples resulted in less accurate diagnoses (Table S3, initial diagnosis).

## Considerations and limitations

### Challenges with treated samples

Samples from treated patients may pose significant challenges to constructing a reliable diagnostic reference. Treatments may alter metabolic profiles, which can misrepresent the untreated disease state. Skewed profiles may resemble other IMDs, potentially leading to misdiagnosis. Therefore, we recommend constructing the reference set using only untreated samples to enhance diagnostic accuracy. Diagnoses or metabolic signature screening involving treated samples should be interpreted cautiously.

### Insufficient samples for refining IMD signatures

A major limitation of this study is the lack of sufficient sample numbers and diversity across IMDs, which limits the algorithm’s ability to refine IMD-specific signatures. The limited sample size also prevents a thorough investigation of potential confounding factors such as age and sex. To mitigate their influence, a large and diverse control group, spanning both sexes and a broad age range, was included. Each patient sample was trained against this heterogeneous control set, reducing the likelihood that age- or sex-dependent features would consistently appear in the final selection. Nevertheless, larger and more diverse datasets are needed to better account for inter-individual variability and identify more robust and specific metabolic signatures for improved differentiation between IMDs.

### Limited metabolite annotation

The number of annotated metabolites in this study is limited due to the stringent selection criteria, and the metabolite coverage may be biased toward specific IMD types. This constraint reduces the algorithm’s ability to generalize across different IMDs. Expanding the range of annotated metabolites without compromising high precision measurements, would create a more comprehensive metabolic signature reference map, improving the algorithm’s diagnostic capabilities across a wider array of IMDs.

## Conclusion

We introduce a novel diagnostic algorithm for IMDs that is designed for small sample sizes while integrating an iterative improvement process. This allows the algorithm to continuously integrate new data and refine IMD-specific metabolic signatures over time, thereby improving diagnostic accuracy and expanding our understanding of IMDs. Its effectiveness was demonstrated across two case studies involving diverse IMDs with a diagnostic accuracy of 87%. With its flexibility to incorporate expert judgment and scalability to accommodate additional metabolites and IMDs, this tool holds significant potential for enhancing clinical diagnostics in IMDs.

## Electronic supplementary material

Below is the link to the electronic supplementary material.

Supplementary Material 1

Supplementary Material 2

Supplementary Material 3

Supplementary Material 4

## Data Availability

No datasets were generated or analysed during the current study.
