# Supplementary material for: A diagnostic algorithm for inherited metabolic disorders using untargeted metabolomics
Source: Metabolomics. 2025 Jul 27;21(4):101. doi: 10.1007/s11306-025-02302-7 (PMC12301266; doi:10.1007/s11306-025-02302-7)
Supplement: Supplementary file 1 — Supplementary Material 1 [file 11306_2025_2302_MOESM1_ESM.docx]

A diagnostic algorithm for inherited metabolic disorders using untargeted metabolomics

Qian Gao^1,*^, Adnan Khan^1^, Mette Christensen^2^, Xiaomin Zhou^2^, Allan Lund^2,3^, Sabine Weller Grønborg^2,3^, Flemming Wibrand^2^, Elsebet Østergaard^2,4^, Thomas Moritz^1,*^

^1^Novo Nordisk Foundation Center for Basic Metabolic Research, University of Copenhagen, Copenhagen, Denmark

^2^Department of Clinical Genetics, Copenhagen University Hospital Rigshospitalet, Copenhagen, Denmark

^3^Center for Inherited Metabolic Diseases, Departments of Pediatrics and Adolescent Medicine and Clinical Genetics, Copenhagen University Hospital Rigshospitalet, Copenhagen, Denmark

^4^Department of Clinical Medicine, University of Copenhagen, Copenhagen, Denmark

*Corresponding author: Thomas Moritz, [thomas.moritz@sund.ku.dk](mailto:thomas.moritz@sund.ku.dk); Qian Gao, [qian.gao@sund.ku.dk](mailto:qian.gao@sund.ku.dk)

# Supplementary materials

**Table S1.** Sample list of Case study 1. The MIM-code refers to the specific entries within the OMIM database. Each entity refers to the particular genetic disorder, used for diagnostics testing in case study 1. More than one MIM-code refers to samples with similar biochemical phenotype but different affected genes.

| **IMD subtype** | **Number of samples** |
| --- | --- |
| Control | 136 |
| Argininosuccinic aciduria (MIM#207900) | 2 |
| Biotinidase deficiency (MIM#253260) | 1 |
| Carbamoylphosphate synthetase deficiency (MIM#237300) | 1 |
| Carnitine transporter deficiency (CTD; MIM#212140) | 2 |
| Citrin deficiency (MIM#605814) | 1 |
| Combined malonic and MMA-aciduria (MIM#614265) | 1 |
| Early-onset vitamin B6-dependent epilepsy (MIM#617290)-1 | 1 |
| Early-onset progressive encephalopathy with brain edema and/or leukoencephalopathy-1 (MIM#617138) | 1 |
| Galactosemia 1 (MIM#230400) | 1 |
| Guanidinoacetate methyltransferease deficiency (MIM#612736) | 1 |
| Holocarboxylase synthase deficiency (HLCSD; MIM#253270) | 3 |
| HMG-CoA lyase deficiency (MIM246450) | 1 |
| D-2-hydroxyglutaric aciduria (MIM#600721) | 2 |
| L-2-hydroxyglutaric aciduria (MIM#236792) | 1 |
| Hyperprolinaemia type 2 (MIM#239510) | 1 |
| Isovaleryl-CoA dehydrogenase deficiency (IVDD: MIM#243500) | 5 |
| Lesch-Nyhan disease (MIM#300322) | 1 |
| Lysinuric protein intolerance (MIM#222700) | 2 |
| Malonyl-CoA decarboxylase deficiency (MIM#248360) | 3 |
| Maple syrup urine disease (MSUD; e.g. MIM#248600 and MIM#620699) | 7 |
| Medium chain acyl-CoA dehydrogenase deficiency (MCADD; MIM#201450) | 17 |
| 3-methylcrotonyl-CoA carboxylase deficiency (MIM#210200 or MIM#210210) | 1 |
| Methylmalonic aciduria (MMA; MIM#251000 and MIM#251110) | 7 |
| Methylmalonic aciduria and homocystinuria (cblC type) (MIM#277400) deficiency | 1 |
| Mitochondrial DNA depletion syndrome 1 (MIM#606341) | 1 |
| Molybdenum cofactor deficiency (MIM#252160) | 1 |
| Multiple acyl-CoA dehydrogenase deficiency (MIM#231680) | 1 |
| Nonketotic hyperglycinemia (MIM#605899) | 2 |
| Peroxisome biogenesis disorders (several MIMs) | 3 |
| Pyruvat carboxylase deficiency (MIM#266150) | 1 |
| Strongly elevated pipecolic acid (IMD unknown) | 1 |
| Succinyl-CoA transferase deficiency (MIM#245050) | 1 |
| Tyrosinemia type 1 (MIM#276700) | 1 |
| Vitamin B12 deficiency (dietary origin) | 1 |

**Table S2.** Cross validation scheme.

|  | Case study 1 | | Case study 2 | |
| --- | --- | --- | --- | --- |
|  | IMD samples | Control samples | IMD samples | Control samples |
| Initial | 35 (35 subtypes) | 5 | 11 (11 subtypes) | 5 |
| 1^st^ iteration | 4 (4 subtypes) | 5 | 10 (10 subtypes) | 5 |
| 2^nd^ iteration | 4 (4 subtypes) | 5 | 9 (9 subtypes) | 5 |
| 3^rd^ iteration | 3 (3 subtypes) | 5 | 8 (8 subtypes) | 5 |
| Test | 31 (13 subtypes) | 5 | 57 (11 subtypes) | 5 |

* The whole procedure was repeated for 100 times with randomly selected samples within each category.

**Table S3.** Diagnosis for 31 samples in case study 1.

Untreated for train (only untreated in start train, the other iterations can contain treated samples)

|  | Rank 1 | Rank 2 | Rank 3 | Total |
| --- | --- | --- | --- | --- |
| Initial | 7.6 (3.1) | 3 (1.5) | 1.7 (1.2) | 12.3 (3.4) |
| 1^st^ iteration | 8.3 (3.2) | 3.1 (1.7) | 1.8 (1.2) | 13.3 (3.6) |
| 2^nd^ iteration | 9.3 (3.1) | 3 (1.5) | 2 (1.4) | 14.4 (3.5) |
| 3^rd^ iteration | 9.6 (3) | 3 (1.7) | 1.9 (1.3) | 14.5 (3.4) |
